# Osteoarthritis genetic risk acting on the galactosyltransferase gene *COLGALT2* has opposing functional effects in articulating joint tissues

**DOI:** 10.1186/s13075-023-03066-y

**Published:** 2023-05-19

**Authors:** Yulia S. Kehayova, J. Mark Wilkinson, Sarah J. Rice, John Loughlin

**Affiliations:** 1 Newcastle University, Biosciences Institute, International Centre for Life, Newcastle Upon Tyne, NE1 3BZ UK; 2 Department of Oncology and Metabolism, University of Sheffield, Sheffield, UK

**Keywords:** Genetics, Epigenetics, DNA methylation, *COLGALT2*, Synovium

## Abstract

**Background:**

Investigation of cartilage and chondrocytes has revealed that the osteoarthritis risk marked by the independent DNA variants rs11583641 and rs1046934 mediate  their effects by decreasing the methylation status of CpG dinucleotides in enhancers and increasing the expression of shared target gene *COLGALT2*. We set out to investigate if these functional effects operate in a non-cartilaginous joint tissue.

**Methods:**

Nucleic acids were extracted from the synovium of osteoarthritis patients. Samples were genotyped, and DNA methylation was quantified by pyrosequencing at CpGs within the *COLGALT2* enhancers. CpGs were tested for enhancer effects using a synovial cell line and a reporter gene assay. DNA methylation was altered using epigenetic editing, with the impact on gene expression determined using quantitative polymerase chain reaction. In silico analysis complemented laboratory experiments.

**Results:**

The rs1046934 genotype did not associate with DNA methylation or *COLGALT2* expression in the synovium, whereas the rs11583641 genotype did. Surprisingly, the effects for rs11583641 were opposite to those previously observed in cartilage. Epigenetic editing in synovial cells revealed that enhancer methylation is causally linked to *COLGALT2* expression.

**Conclusions:**

This is the first direct demonstration for osteoarthritis genetic risk of a functional link between DNA methylation and gene expression operating in opposite directions between articular joint tissues. It highlights pleiotropy in the action of osteoarthritis risk and provides a cautionary note in the application of future genetically based osteoarthritis therapies: an intervention that decreases the detrimental effect of a risk allele in one joint tissue may inadvertently increase its detrimental effect in another joint tissue.

**Supplementary Information:**

The online version contains supplementary material available at 10.1186/s13075-023-03066-y.

## Background

Statistical, in silico and laboratory-based functional fine-mapping studies enable the identification of the gene targets of DNA variants that associate with common polygenic diseases [1–4]. These investigations also highlight the cell and tissue type in which genetic risk is operating [1–4]. Such molecular and cellular insights are essential preludes to clinically translating genetic discoveries [1–4]. A range of functional tools have been employed to mechanistically link osteoarthritis (OA) risk alleles with target genes [5, 6]. Through these investigations, it has become apparent that changes in the DNA methylation status of CpG dinucleotides neighbouring an OA-associated variant can act as a direct functional intermediate between allele and target: the risk allele associates with a change in CpG methylation which alters target gene expression [6, 7].

Recent examples of this are two independent OA-associated variants that both impact the expression of *COLGALT2* via CpGs located in enhancers [8, 9]. *COLGALT2* encodes procollagen galactosyltransferase 2, an enzyme that glycosylates collagens and proteins with collagen domains [10]. This post-translational modification alters the biological activity of a tissue’s extracellular matrix (ECM). For example, over-glycosylation reduces collagen fibril diameter and lessens the ability of a tissue to withstand mechanical load [11]. The OA-associated variants investigated were rs11583641 (C > T), located in the 3′ untranslated region (3′UTR) of *COLGALT2*, and rs1046934 (A > C), located in *TSEN15*, downstream of *COLGALT2* on chromosome 1 [8, 9]. The variants are in near-perfect linkage equilibrium, with pairwise *r*^2^ of 0 and *D*′ of 0.08 in European ancestry cohorts. Genotype at rs11583641 associates with the methylation of cg18131582 and flanking CpGs in cartilage, forming a methylation quantitative trait locus (mQTL) at an enhancer within *COLGALT2* intron 10 (of 11) [8]. Genotype at rs1046934 forms a cartilage mQTL with cg15204595, located in an enhancer within intron 1 of *COLGALT2*, and with cg21606956, located in a downstream intergenic enhancer [9]. Functional studies demonstrated that for both variants, the OA risk alleles (C for rs11583641, A for rs1046934) associate with reduced methylation levels of the CpGs and with increased *COLGALT2* expression, with epigenetic editing demonstrating that methylation regulates the change in expression [8, 9].

These *COLGALT2* experiments focussed on articular cartilage and the cell responsible for synthesising this tissue, the chondrocyte [8, 9]. One cause of cartilage loss in OA is the increased expression by chondrocytes of collagenases and aggrecanases, leading to a breakdown of collagen and a loss of aggrecan from the cartilage ECM [12, 13]. Because of its centrality to the disease process, most investigations prioritise the study of this tissue [12, 13]. OA pathology is not however restricted to the cartilage, with changes observed in other tissues of the articulating joint [14–16]. A non-cartilaginous tissue that has received a relatively high degree of attention is the synovium [14, 17, 18]. This tissue lines the joint capsule and produces synovial fluid that lubricates the articulating surface and supplies oxygen and nutrients to the avascular cartilage [17]. During OA, the synovium can become inflamed and fibrotic [17]. Like the cartilage, the synovium can be readily accessed following joint replacement surgery, permitting the isolation of cells and the extraction of nucleic acids that can be used in molecular studies [18].

In this report, we set out to determine whether the mQTL and *COLGALT2* expression effects that had been observed in cartilage [8, 9] were detectable in synovium. We used a range of molecular approaches complemented by in silico studies. We investigated the nucleic acids extracted from synovial tissue donated by OA patients undergoing knee arthroplasty and utilised a synovial cell line for functional analysis, including precision epigenome editing.

## Methods

### Patient samples

Synovium samples were obtained from 88 patients undergoing joint arthroplasty at the Newcastle upon Tyne NHS Foundation Trust hospitals for primary knee OA. Ethical approval was granted by the NHS Health Research Authority with each donor providing verbal and written consent (REC reference number 19/LO/0389). Patient details are available in Additional file 1. The patients who provided the synovium samples were separate from the patients who provided the cartilage samples that we had previously investigated at these loci [8, 9]. The two groups of patients were however recruited from the same northeast England population, using the clinical criteria of primary knee OA disease status, and via the same orthopaedic clinics at the trust hospitals. The two groups have near identical average age at surgery (67.0 years [synovium] versus 66.5 years [cartilage]) and sex distribution (42.0% male [synovium] versus 41.5% male [cartilage]) demographics (Additional file 1). Synovium samples were ground frozen using a Mixer Mill MM 400 (Retsch) and the nucleic acids extracted using an E.Z.N.A DNA/RNA isolation kit (Omega Bio-tek).

### Genotyping

Each DNA variant was PCR amplified using genomic DNA isolated from the synovium. Samples were then genotyped using the PyroMark Q24 Platform (Qiagen), according to the manufacturer’s instructions. Primer sequences were generated by the PyroMark Assay Design Software (Qiagen) and purchased from Integrated DNA Technologies (IDT). Primer sequences are listed in our previous publications [8, 9].

### Methylation quantification

Genomic DNA (500 ng) was treated with sodium bisulfite using EZ DNA Methylation Kits (Zymo Research). Pyrosequencing was used to quantify methylation at the rs11583641 and rs1046934 CpGs, using the assays and protocol described previously [8, 9]. Each measurement was performed in duplicate, and replicate values that differed by > 5% were excluded from the analysis.

### Allelic expression imbalance (AEI) analysis of *COLGALT2*

For the rs11583641 locus, allelic imbalance was measured using this *COLGALT2* 3′UTR transcript variant. For the rs1046934 locus, since this variant does not reside within *COLGALT2*, the *COLGALT2* 5′UTR variant rs114661926 (C > G) was used (*r*^2^ of 0.79 with rs1046934 in European ancestry cohorts; LDlink, https://ldlink.nci.nih.gov). The relative ratio of alleles was quantified using pyrosequencing in DNA and complementary DNA (cDNA) from heterozygous patients, using the assays and protocol described previously [8, 9]. Synovium samples were analysed in triplicate, with replicate values having < 5% difference.

### Reporter gene assay

To investigate the region harbouring cg18131582 and its flanking CpGs, we used the same Lucia CpG-free promoter vector (InvivoGen) clones that we had used previously [8]. These were methylated or mock-methylated [8] and transfected into SW982 cells, a human synovial cell line (ATCC). Transfections were performed with 100 ng of pCpG-free promoter and 10 ng of pGL3-promoter (Promega) vectors using Lipofectamine 2000 (Invitrogen). Cells were lysed after 24 h. Luminescence was read and analysed, as described previously [8].

### Epigenetic modulation using dead Cas9 (dCas9)

Six CRISPR guide RNA (gRNA) sequences were used to alter the methylation status of cg18131582 and its flanking CpGs in the SW982 cell line. These are the six gRNAs used previously, where they were labelled gRNA3-gRNA8 [8]. Here, they are labelled gRNA1-gRNA6. Methylation and demethylation were performed using dCas9-DNMT3a and dCas9-TET1 plasmids, respectively, with catalytically inactive DNMT3a and TET1 as controls, as described previously [9]. Plasmid DNAs were transfected into the SW982 cells by nucleofection (Lonza). Gene expression was measured by real-time quantitative PCR (RT-qPCR) (QuantStudio 3) using the TaqMan primers and probes described previously [8]. The expression of *COLGALT2* was measured relative to that of the housekeeping genes *18S*, *GAPDH*, and *HPRT1*, using the formula 2^−Δct^ [19].

### In silico analysis

LDlink (https://ldlink.nci.nih.gov) and European ancestry cohort data were used to determine linkage disequilibrium values between DNA variants and to calculate haplotype frequencies. JASPAR [20] and the UCSC Genome Browser (https://genome.ucsc.edu/; hg19) were used to identify and visualise transcription factors predicted to bind at or close to cg18131582 and its flanking CpGs. To assess if these transcription factors were expressed in cartilage or synovium, we investigated bulk RNA sequencing data generated from the cartilage chondrocytes of 10 OA patients and from the synovium synovial fibroblasts (fibroblast-like synoviocytes) of 10 OA patients [21, 22] (Gene Expression Omnibus [GEO; https://www.ncbi.nlm.nih.gov/geo/] accession numbers GSE111358 and GSE112658).

### Statistical analysis

For graphical representations of DNA methylation data, methylation status was plotted in the form of *β*-values, ranging from 0 (no methylation) to 1 (100% methylation). For statistical analysis of methylation data, *β*-values were converted to *M*-values [23]. In mQTL analysis, linear regression was used to assess the relationship between CpG methylation and genotype (0, 1 or 2 copies of the minor allele). The Mann–Whitney *U* test was used to calculate *P* values when comparing methylation levels irrespective of genotype. Wilcoxon matched-pairs signed rank test was used to calculate *P* values in AEI analysis. For Lucia reporter gene assays, *P* values were calculated by paired and unpaired t-tests with Holm-Šídák correction. Paired *t*-tests were used to calculate *P* values for changes in gene expression following epigenetic modulation. Statistical tests were performed in GraphPad Prism.

## Results

### Investigation of OA-associated mQTLs in the synovium

**Fig. 1:**
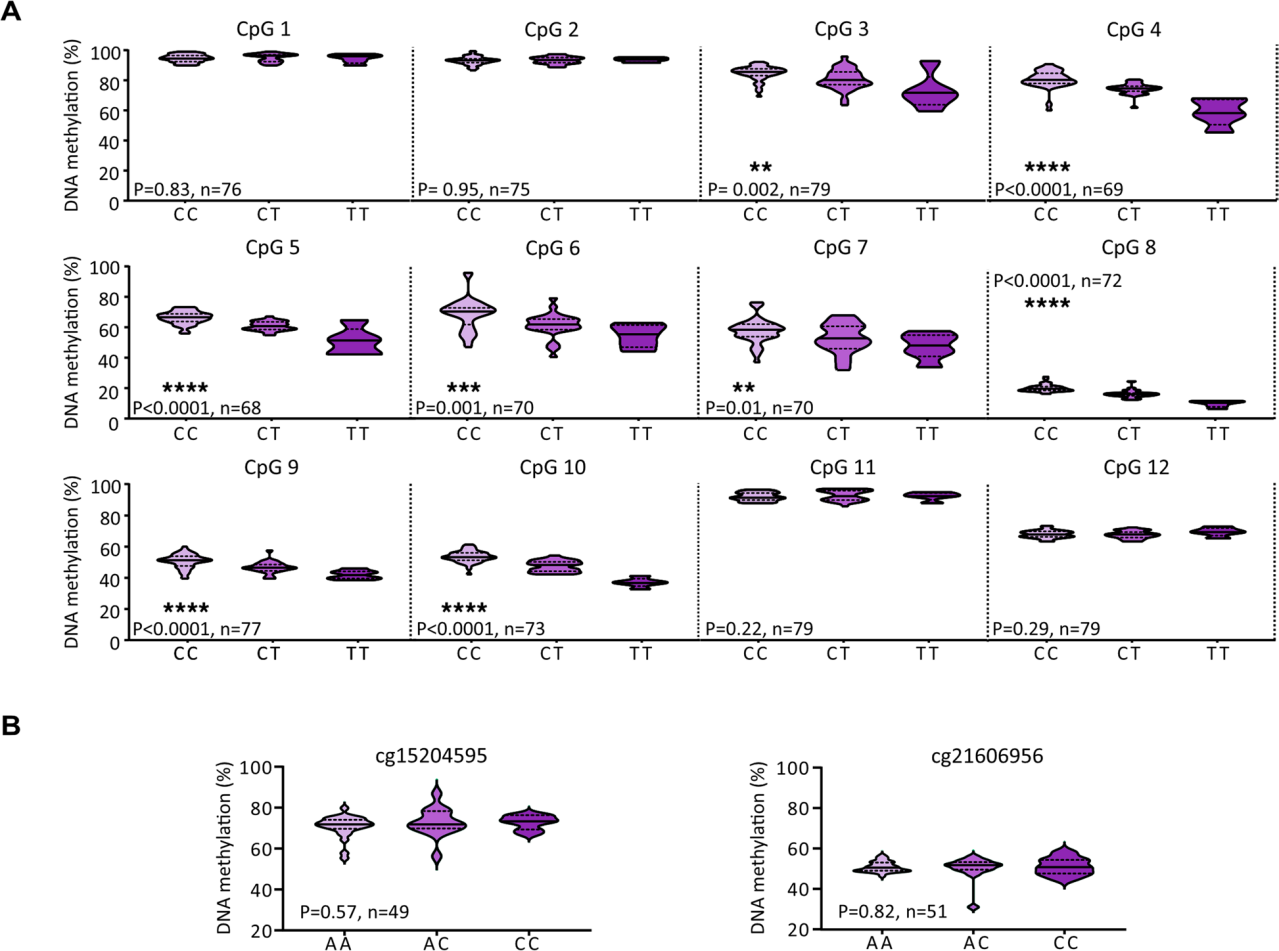
Methylation quantitative trait locus (mQTL) analysis. Violin plots show the DNA methylation values at synovium samples (*n* = 49–79 patients; differences in numbers due to a variable number of patient samples per CpG passing quality control) for **A** cg18131582 (CpG9) and its 11 flanking CpGs stratified by genotype at rs11583641 and for **B** cg15204595 and cg21606956 stratified by genotype at rs1046934. Solid and dashed horizontal lines represent the median and interquartile range. *P* values were calculated by linear regression. Significant *P* values (*P* < 0.05) are marked: **P < 0.01; ***P < 0.001; ****P < 0.0001

We quantified methylation at cg18131582 and its 11 flanking CpGs, and at cg15204595 and cg21606956, stratifying the data by genotype at rs11583641 and rs1046934, respectively (Fig. 1 and Additional files 2 and 3). Significant mQTLs (*P* < 0.05) were identified at cg18131582 (CpG9) and 7 of its flanking CpGs (Fig. 1A). The four most distal CpGs (CpGs 1, 2, 11 and 12) were not significant, defining the limit of the differentially methylated region (DMR). At the 8 significant CpGs, the risk allele, C, of rs11583641 was associated with higher levels of DNA methylation relative to the non-risk allele, T (Fig. 1A). This is the opposite of what we observed in cartilage [8]. Furthermore, in cartilage, the DMR extended only between CpGs 8 and 10 [8]. Significant mQTLs were not identified at cg15204595 or cg21606956 (both *P* > 0.05, Fig. 1B).

**Fig. 2:**
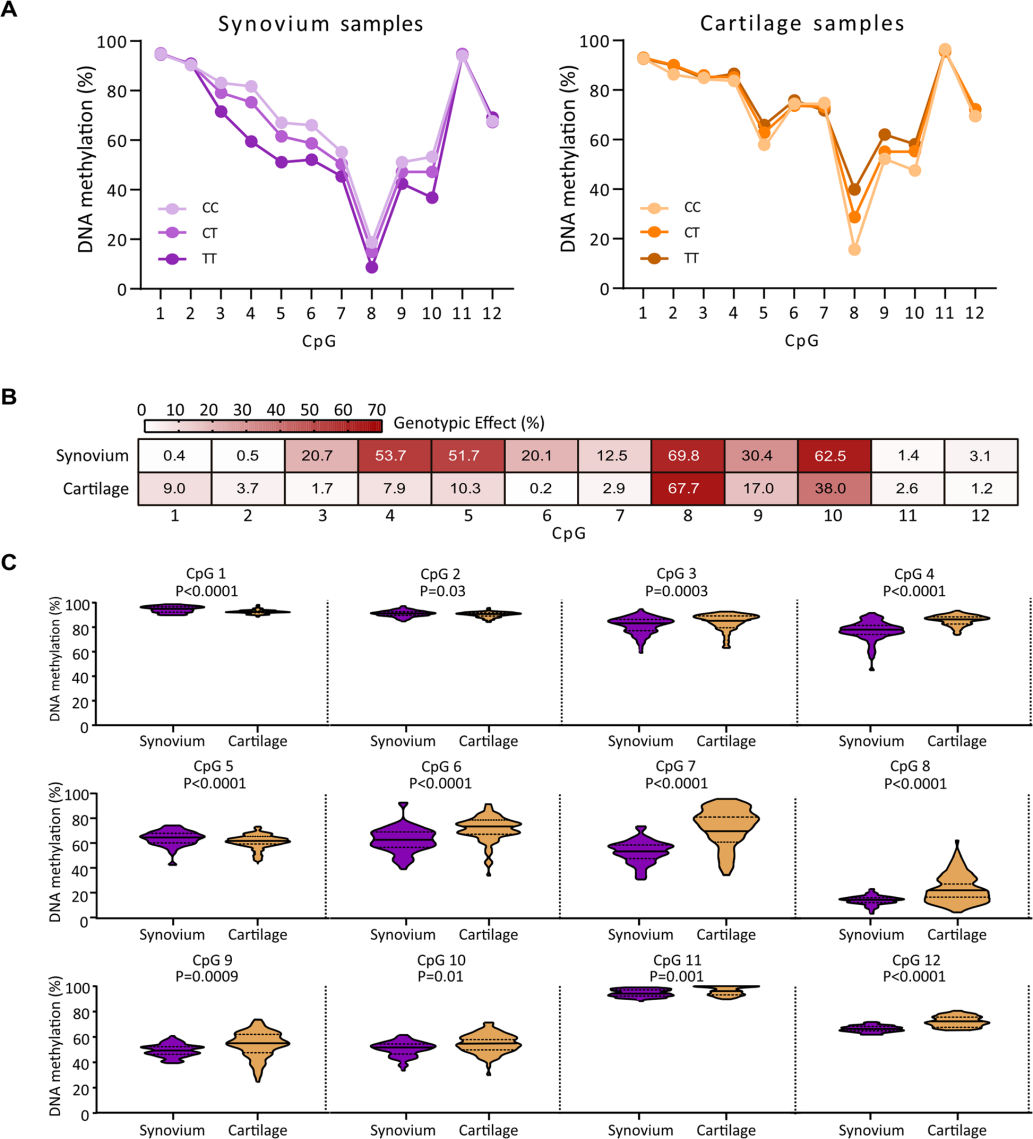
Comparison of the rs11583641 mQTL between synovium and cartilage. **A** The median DNA methylation value for each of the 12 CpGs stratified by genotype at rs11583641. **B** Heatmap showing the contribution of rs11583641 genotype to DNA methylation at the 12 CpGs. *r*^2^ values calculated by linear regression and expressed as a percentage. **C** DNA methylation at each CpG stratified by tissue type and irrespective of genotype at rs11583641. *P* values were calculated by the Mann–Whitney *U* test

We analysed the median DNA methylation levels of cg18131582 and its 11 flanking CpGs and compared to OA cartilage DNA methylation levels from our previous study [8] (Fig. 2A). The pattern of DNA methylation across the region was comparable between the two tissues, with hypermethylation at CpGs 1–4 and CpG11 (median DNA methylation > 75%) and hypomethylation at CpG8 (median DNA methylation < 25%). We next quantified the rs11583641 genotypic effect (expressed as %) on methylation at cg18131582 and its flanking CpGs (Fig. 2B). For 7 of the 8 DMR CpGs (CpGs 3–7, 9 and 10), a larger genotypic effect was observed in the synovium compared to the cartilage whilst for CpG8, the effects were comparable (69.8% in the synovium, 67.7% in the cartilage). We then compared the CpG methylation levels between the synovium and the cartilage unstratified by rs11583641 genotype (Fig. 2C). There were significant differences in methylation levels between the two tissues for all 12 CpGs. At 9/12 CpGs, the median methylation levels measured in the synovium were lower compared to those measured in the cartilage, with the greatest difference measured at CpG7 (21.7%). Our synovium patients range in age from 49 to 88 years. There was no association between age and synovium methylation levels at any of the 12 CpGs (Additional files 4 and 5).

### Genotype at rs11583641 associates with *COLGALT2* expression in synovium

**Fig. 3:**
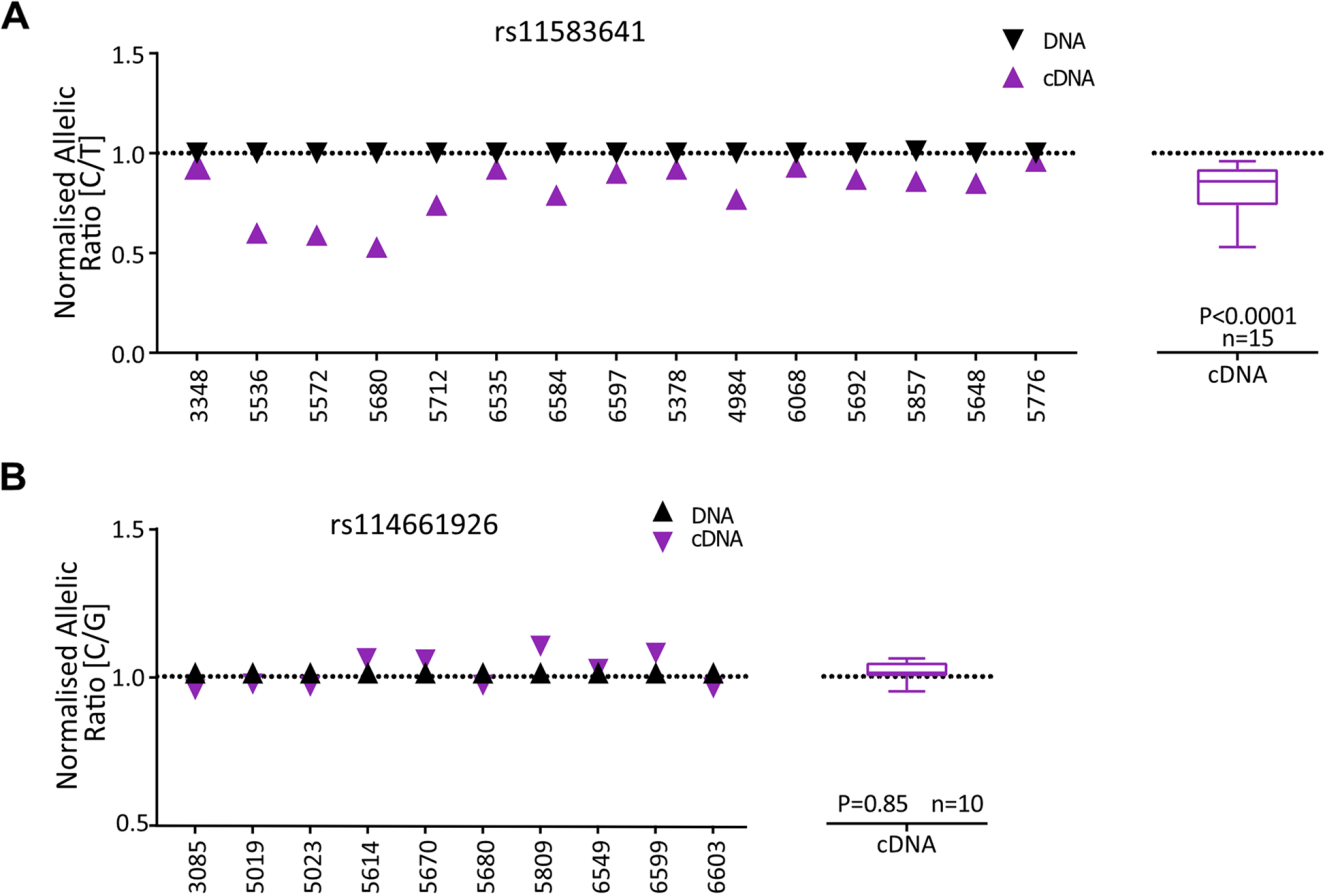
Allelic expression imbalance (AEI) analysis. **A** Left, allelic (C/T) ratios in the synovium samples from knee OA patients heterozygous for rs11583641 (*n* = 15, numbers on the *x*-axis are patient sample IDs). In each sample, the ratio of values for cDNA and DNA between the C (OA risk) allele and T allele was plotted; each symbol represents the average of three technical repeats. Right, the mean DNA and cDNA values in the presence of the C allele versus the T allele in the 15 heterozygous patients. Values are shown as a box plot, with the lines inside the box representing the median, the box showing the interquartile range and the whiskers showing the minimum and maximum values. **B** Left, allelic (C/G) ratios in the synovium samples from knee OA patients heterozygous for rs114661926 (*n* = 10). In each sample, the ratio of values for cDNA and DNA between the C (OA risk) allele and G allele was plotted; each symbol represents the average of three technical repeats. Right, the mean DNA and cDNA values in the presence of the C allele versus the G allele in the 10 heterozygous patients. Values are shown as a box plot, as above. *P* values were calculated by Wilcoxon matched-pairs signed rank test

We next investigated the effects of the rs11583641 and rs1046934 genotypes on allelic expression of *COLGALT2* in the synovium (Fig. 3 and Additional file 6). For rs1046934, we used the *COLGALT2* transcript variant rs114661926 (*r*^2^ = 0.79 in European ancestry cohorts).

In individuals heterozygous for rs11583641, there was a significant (*P* < 0.0001) imbalance between the C and T transcripts of *COLGALT2* (Fig. 3A). A 0.19-fold mean decrease in the expression of the OA risk allele, C, of rs11583641 was observed. This is opposite to the cartilage, where the risk allele showed increased expression [8]. We did not detect an allelic imbalance between the C and G transcripts of *COLGALT2* in rs114661926 heterozygous patients (Fig. 3B).

Our DNA methylation and *COLGALT2* expression analysis in the synovium revealed an association with the rs11583641 genotype but not with the rs1046934 genotype. For subsequent investigations in this study, we chose to focus solely on the functional risk of OA driven by the rs11583641 locus.

### The cg18131582 DMR is a methylation-sensitive enhancer in synovial cells

**Fig. 4:**
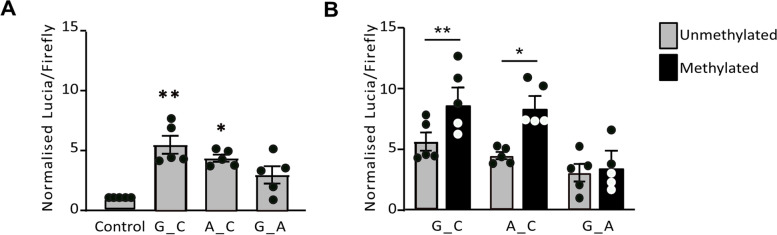
Investigation of enhancer activity at the DMR region in SW982 synovial cells. **A** Lucia reporter assays assessing the enhancer activity in the presence of constructs containing the 3 haplotypes of rs943409 and rs734657 and **B** the 3 haplotypes in a methylated or unmethylated state; values were normalised to those in an empty vector control. Symbols represent individual samples (*n* = 5 per group). Bars show the mean ± SEM. *P < 0.05; **P < 0.01, calculated by paired *t*-test with Holm-Šídák correction for empty control versus construct or by unpaired *t*-test with Holm-Šídák correction for unmethylated construct versus methylated construct

We next tested the region containing cg18131582 and its flanking CpGs for regulatory activity using a reporter gene assay in the synovial cell line SW982 (Fig. 4 and Additional file 7). We focussed on the 8 CpGs within the cg18131582 DMR, at which significant mQTLs were observed. A 503-bp region encompassing CpGs 3–10 was cloned into a Lucia reporter gene vector. This region also contains the DNA variants rs943409 (G > A) and rs734657 (C > A). rs943409 is not in linkage disequilibrium (LD) with rs11583641 (*r*^2^ = 0.09) whereas rs734657 is (*r*^2^ = 0.70), with the OA risk allele, C, of rs11583641 nearly always occurring on a chromosome containing allele C of rs734657. rs943409 and rs734657 form 3 naturally occurring haplotypes in Europeans: G_C (53.6%), G_A (26.7%) and A_C (19.7%). All 3 were tested for their impact on enhancer activity. Two of the 3 constructs, both of which contain allele C of rs734657 (G_C and A_C), had significantly increased enhancer activity (*P* = 0.0076 and *P* = 0.016, respectively) (Fig. 4A). There was no difference in enhancer activity between these two constructs (*P* > 0.05). Methylation of the enhancer region significantly increased the enhancer activity of the G_C and A_C constructs (*P* = 0.0044 and *P* = 0.01, respectively) (Fig. 4B). This reporter gene assay data suggests that the cg18131582 DMR acts as a methylation-sensitive enhancer in synovial cells and that rs734657 genotype impacts this enhancer function. This agrees with the data from our report using the chondrocyte cell line Tc28a2, although in that study DNA methylation decreased enhancer activity of the G_C and A_C constructs [8].

### Altering enhancer methylation alters *COLGALT2* expression

**Fig. 5:**
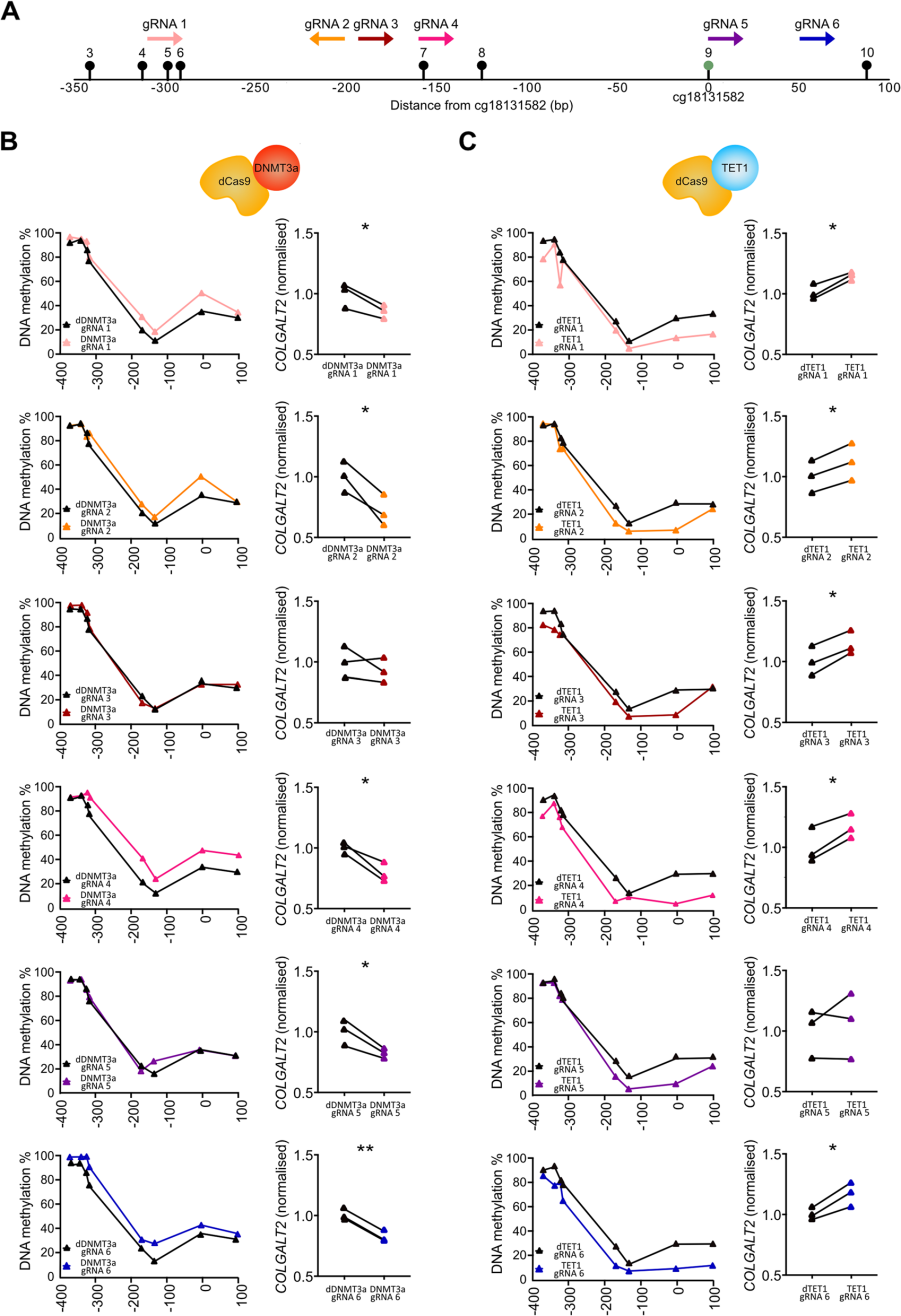
Epigenetic modulation of the enhancer in SW982 synovial cells. **A** Schematic diagram showing the genomic position of the 6 guide RNAs (gRNAs) used for modulating DNA methylation (gRNAs 1–6), relative to the 8 CpGs of the DMR; cg18131582 is CpG9 (green circle). **B** Left, DNA methylation levels at the 8 CpGs in SW982 synovial cells following the expression of dead DNMT3A (dDNMT3a)-dead Cas9 (dCas9) protein in controls (black line) or in samples with active DNMT3A (DNMT3a)-dCas9 protein, each with targeting gRNAs (each *n* = 3). Right, *COLGALT2* expression in SW982 synovial cells following editing of DNA methylation with gRNAs. Values were normalised to the mean values of control cells (each *n* = 3). **C** Left, DNA methylation levels at the 8 CpGs in SW982 synovial cells following expression of dead TET1 (dTET1)-dCas9 protein in controls (black line) or in samples with active TET1 (TET1)-dCas9 protein, each with targeting gRNAs (each *n* = 3). Right, *COLGALT2* expression in SW982 synovial cells following editing of DNA methylation with gRNAs. Values were normalised to the mean values of control cells (each *n* = 3). *P < 0.05; **P < 0.01, calculated by paired *t*-test

We then investigated whether altering the DNA methylation status of the enhancer within its natural genomic context impacts *COLGALT2* expression (Fig. 5 and Additional files 8 and 9). We used dCas9 modulators of CpG methylation in the SW982 synovial cells: dCas9-DNMT3a to methylate the CpGs or dCas9-TET1 to demethylate the CpGs. Six gRNAs (gRNAs 1–6) were used to target DMR CpGs 3–10 (Fig. 5A). We expressed individual gRNAs in the SW982 cells along with the dCas9 constructs. DNA methylation was successfully increased using 5 of the 6 gRNAs (gRNAs 1–2 and 4–6) (Fig. 5B, left) and successfully decreased using all 6 gRNAs (Fig. 5C, left). The increases in DNA methylation resulted in significant (*P* < 0.05) decreases in *COLGALT2* expression, whereas the decreases in DNA methylation resulted in significant increases in *COLGALT2* expression (Fig. 5B, right, Fig. 5C, right). This is consistent with the results from our epigenetic modulation of Tc28a2 chondrocyte cells [8].

In the dCas9-DNMT3a experiment, gRNA5 increased the methylation only of CpG8 but this still resulted in a significantly decreased expression of *COLGALT2* (Fig. 5B). Relative to the other DMR CpGs, CpG8 is hypomethylated and has the largest genotypic effect in the synovium and in the cartilage (Fig. 2). These results indicate that the methylation status of CpG8 may be particularly important to the function of the enhancer.

### Transcription factors are predicted to bind to the enhancer

**Fig. 6:**
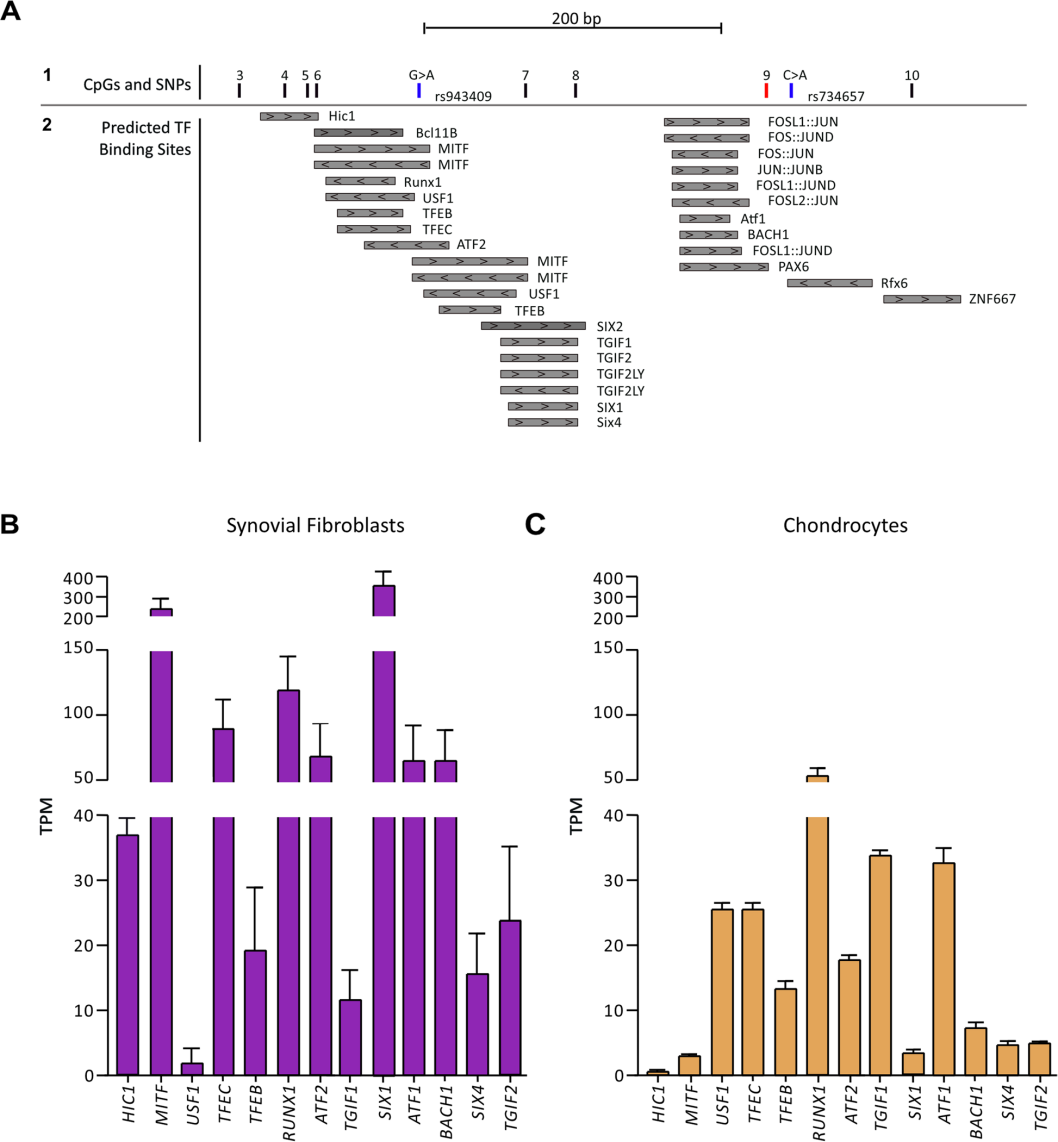
Transcription factors (TFs) predicted to bind within the DMR. **A** Section 1,  location of the 8 DMR CpGs; cg18131582 is CpG9 (red line). Also shown are the locations of the DNA variants (blue bars) rs943409 (G > A) and rs734657 (C > A). Section 2, TF-binding sites as predicted by JASPAR and visualised in the UCSC Genome Browser (hg19). The TFs are marked by grey bars with the direction of the arrows within the boxes indicating the DNA strand the TF is predicted to bind to (arrows pointing to the left = antisense strand,  arrows pointing to the right = sense strand). **B**, **C** Expression levels (transcripts per million (TPM)) of the TFs in synovium synovial fibroblasts from OA patients (*n* = 10) and in cartilage chondrocytes from OA patients (*n* = 10). Bars show the mean ± SEM. The *y*-axes are linear segmented scales with 3 segments

The reporter gene assay and dCas9 experiments imply that altering the DNA methylation status of the DMR has effects on the functioning of the enhancer and on gene expression. A potential mechanism for this could be an alteration of the binding efficiency of transcription factors to DNA that can occur in response to methylation changes [24–26]. If this were the mechanism by which the DMR regulates enhancer activity, we would expect the CpGs to be part of, or physically close to, transcription factor binding sites. To assess this, we used JASPAR [20] and identified 20 transcription factors (including AP1 FOS/JUN dimers) predicted to bind at or near the 8 DMR CpGs (Fig. 6A). Many of these are expressed in synovial fibroblasts (Fig. 6B) and chondrocytes (Fig. 6C) from OA patients. Variant rs734657, whose alleles differentially impacted enhancer function in the reporter gene assay (Fig. 4), is located 13 bp from cg18131582 (CpG9) (Fig. 6A). rs734657 and cg18131582 are not predicted to share a binding site with a transcription factor (Fig. 6A). rs734657 is predicted to be part of the broad binding sequence for Rfx6 (Fig. 6A), but it is not within the core motif consensus for Rfx6 binding: this motif consensus is CCTAGCAAC, and the sequence at the site is CCTAGCAAT, with rs734657 being the base immediately preceding this sequence.

## Discussion

We have shown that a common OA risk DNA variant has opposite functional effects on its target gene between synovium and cartilage. The risk allele, C, of rs11583641 associates with increased enhancer methylation and decreased *COLGALT2* expression in the synovium but with decreased enhancer methylation and increased *COLGALT2* expression in the cartilage [8]. We have previously reported on several OA risk variants that show an association with gene expression and/or with methylation levels of CpGs in cartilage [27–32]. For many of these, we have also investigated the synovium, and for some, we observed a common effect of risk allele on DNA methylation, which then has opposing effects on gene expression [29–31]. For example, the risk allele, A, of OA variant rs75621460 associates with increased DNA methylation of a *TGFB1* enhancer in the synovium and cartilage, resulting in decreased *TGFB1* expression in synovium but increased *TGFB1* expression in cartilage [31]. These earlier results highlight the interplay between genetic variants and DNA methylation in OA and show how tissue-specific effects on transcription can result from changes to the DNA methylation status of an enhancer. Our new report adds an additional level to this interplay, by revealing that an OA risk allele can have opposing effects on both enhancer methylation and gene expression within different tissues of the articulating joint. Future studies should aim to investigate multiple joint tissues collected simultaneously from patients to assess the degree to which OA risk variants have joint-wide or tissue-specific functional effects.

**Fig. 7:**
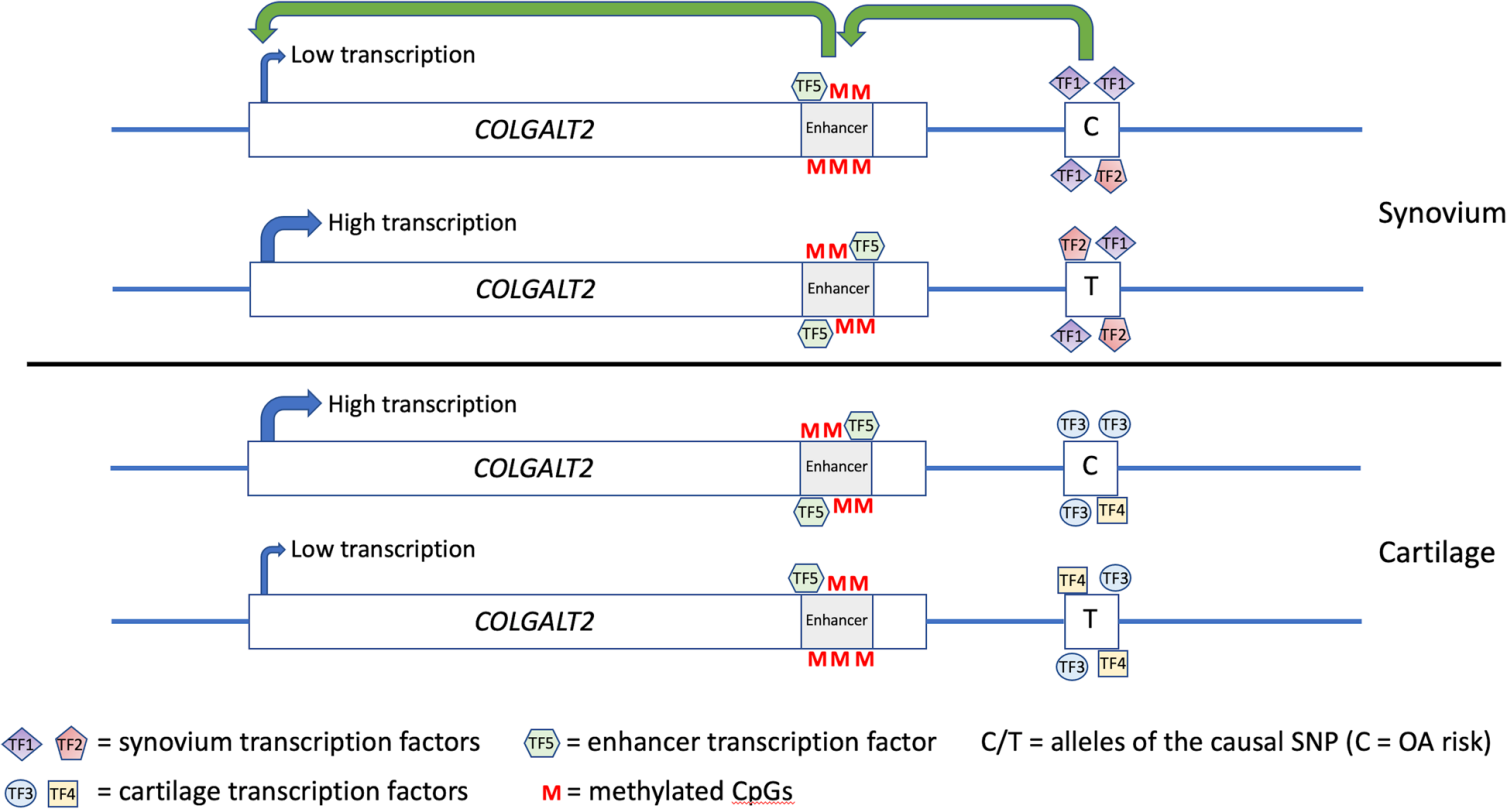
Model accounting for the opposite effects between synovium and cartilage of the risk allele on enhancer methylation and *COLGALT2* expression. To be read in conjunction with Additional file 10. The causal variant has hypothetical alleles C (risk) and T (non-risk). In the synovium, the alleles differentially bind transcription factors TF1 and TF2; in the cartilage, they differentially bind transcription factors TF3 and TF4. Differential transcription factor binding leads to allele-specific enhancer methylation, resulting in quantitative differences in the binding of a common transcription factor (TF5). Low levels of bound TF5 lead to low levels of *COLGALT2* transcription. In the synovium, allele C is more methylated at the enhancer than allele T, resulting in less TF5 binding to, and therefore relatively low transcription of, allele C. The opposite occurs in the cartilage. This matches our patient’s DNA methylation and *COLGALT2* expression data. The model predicts that for both tissues, decreased enhancer methylation increases *COLGALT2* expression and vice versa. This matches our epigenetic editing data of synovial cell line SW982 (this report) and of chondrocyte cell line Tc28a2 [8]

The relationships between rs11583641 genotype and DNA methylation levels between synovium and cartilage are directly opposing. However, the relationship between methylation and gene expression is consistent between the tissues. In our patient samples, higher methylation levels associated with lower *COLGALT2* expression in the synovium (this study), whilst lower methylation levels associated with higher *COLGALT2* expression in the cartilage (our previous study) [8]. This observation in human patient samples was corroborated by the dCas9 epigenetic editing in the synovium (SW982; this study) and cartilage (Tc28a2; our previous study) [8] cell models. The enzymes responsible for actively methylating and de-methylating DNA (DNMT and TET, respectively) can specifically catalyse the addition or removal of methyl groups from cytosine bases when recruited to genomic sites by other DNA-binding protein complexes, namely transcription factors [24–26]. Such CpGs can be distal from the recruiting DNA sequence, with physical interactions brought about through long-range chromatin looping [26]. Based upon our studies of the *COLGALT2* rs11583641 locus in multiple joint tissues, we hypothesise that in the synovium and cartilage, the transcription factors binding to the site of the functional variant, which can then in turn recruit DNMT3a or TET1 to the region, are distinct (Fig. 7 and Additional file 10, A-C). There are qualitative and quantitative differences in the transcription factors expressed between the synovium and cartilage, and significant transcriptomic differences between the two tissues have been reported [33]. In both tissues, the distinct proteins binding at the causal variant can physically interact with the *COLGALT2* enhancer and thus the CpGs of the DMR (Additional file 10, C). This results in a differential impact of the variant upon enhancer methylation between the tissues (Additional file 10, C). However, in both tissues, methylation of the enhancer results in a decrease in *COLGALT2* expression, and vice versa (Additional file 10, D and E). We hypothesise that this is because methylation of the enhancer precludes transcription factor binding in this region, and the proteins binding at the DMR are expressed in both tissues (Additional file 10, D).

Kreitmaier and colleagues [34] recently published a genome-wide methylation array and genotype array analysis of DNA extracted from the synovium and cartilage of OA patients. They replicated the cartilage cg18131582 mQTL that we had previously reported [8], by using variant rs10797923 (*r*^2^ of 0.81 with rs11583641). They did not report a synovium cg18131582 mQTL. They compared mQTL effects between the synovium and intact cartilage (equivalent to the cartilage we have investigated) across the genome and reported that most of the synovium-cartilage mQTL pairs showed an effect in the same direction, with only 33/143,258 mQTL pairs showing an effect in the opposite direction [34]. Our observation of an opposite effect between the synovium and cartilage at the cg18131582 mQTL is therefore uncommon. Using Mendelian randomisation, Kreitmaier and colleagues [34] went on to highlight cg18131582 as having a potential causative effect in OA. This supports our observation of a mechanistic and potentially causal link between DNA methylation at the DMR, *COLGALT2* expression and increased OA susceptibility.

In our comparison of the genotypic effect of rs11583641 on the *COLGALT2* mQTL, the effects were stronger in the synovium than in the cartilage. We have observed this previously for OA mQTLs reported proximal to the target genes *RWDD2B* and *TGFB1* [30, 31]. However, at an OA risk locus harbouring the gene *PLEC*, encoding the cytoskeletal protein plectin, the mQTL effect sizes were comparable between the two tissues [29]. It is becoming increasingly apparent that OA risk loci can mediate functional effects on non-cartilaginous tissues and that tissues such as the synovium may in fact be impacted at least as much, if not more so, than the cartilage [34–36]. Molecular studies therefore add further support to the growing consensus that initiators of OA, including genetic susceptibility, act on multiple tissues of the articulating joint [37].

As noted earlier, *COLGALT2* encodes procollagen galactosyltransferase 2, an enzyme that post-translationally glycosylates collagens and proteins with collagen domains [10]. It has been proposed that over-glycosylation results in lower collagen molecule cross-links and a less stable collagen fibril [11]. Since the OA risk allele of rs11583641 associates with increased *COLGALT2* expression in the cartilage, the effect may be one of collagen over-glycosylation, resulting in a cartilage tissue less able to withstand mechanical load. Unlike the cartilage, the synovium’s role is not to withstand mechanical load but to synthesise synovial fluid components to lubricate the articular surface [38]. The synovium also provides nutrients to the cartilage, clears debris from the synovial fluid following minor injury and resolves joint inflammation resulting from bacterial or viral infection [38]. Studies of liver injury and liver inflammation suggest that procollagen galactosyltransferase 2 can regulate immune cells and the levels of pro- and anti-inflammatory cytokines via altering the collagen glycosylated state of the liver ECM [39, 40]. Overall, we hypothesise that the OA risk allele at the rs11583641 locus is mediating pleiotropic effects on joint tissues: in the cartilage, it leads to increased procollagen galactosyltransferase 2 activity that compromises collagen fibril integrity and cartilage resilience; in the synovium, it leads to decreased procollagen galactosyltransferase 2 activity, which compromises the response of the tissue to injury and inflammation, resulting in fibrosis.

## Conclusions

We have previously observed that an OA risk allele can associate with the same epigenetic change at an enhancer, which then elicits opposite effects in the expression of a target gene [31]. Until now, we had not observed an OA risk allele that associates with opposing effects on both enhancer DNA methylation and gene expression. Our past and current observations highlight the complex pleiotropy of OA genetic risk, with a risk allele-mediating effects in multiple joint tissues, sometimes in opposing directions, and potentially via different biological pathways. This has implications for any direct future use of OA genetic data for therapeutic intervention. Using the rs11583641 OA association as an example, a small-molecule intervention applied joint-wide (for example, to the synovial fluid) to counteract the increased cartilage expression of *COLGALT2* mediated by the risk allele could lower the expression of the gene in the synovium, the opposite to what is required in that tissue. If there is to be efficient therapeutic exploitation of OA genetic insights [41], our study emphasises the need to delve deeply at a molecular level into the functionality of this genetic risk and to investigate multiple joint tissues and relevant cell types.

## Supplementary Information

**Additional file 1.** Details of the knee OA patients who donated synovium samples for this study, and of the knee OA patients who donated cartilage samples in our previous analysis of the rs11583641 and rs1046934 loci [8, 9].

**Additional file 2. **Methylation data (β-values and M-values) used for rs11583641 mQTL analysis. CpG exclusion rate (text in red) is the percent of genotyped samples that failed QC (replicate values differed by >5%).

**Additional file 3. **Methylation data (β-values and M-values) used for rs1046934 mQTL analysis. CpG exclusion rate (text in red) is the percent of genotyped samples that failed QC (replicate values differed by >5%).

**Additional file 4. **Age versus methylation. Linear regression was used to test for association between age at surgery inyears and DNA methylation levels at cg18131582 (CpG9) and its 11 flanking CpGs. DNAm, DNA methylation. Each dot is data from one individual.

**Additional file 5. **Age versus DNA methylation at the rs11583641 locus.

**Additional file 6.** Allelic expression imbalance (AEI) data.

**Additional file 7. **Lucia reporter gene data.

**Additional file 8. **dCas9 epigenetic modulation (β-values).

**Additional file 9. **RT-qPCR data following dCas9 epigenetic modulation.

**Additional file 10. **Flow version of Fig. 7. (**A**) The causal variant has hypothetical alleles C (risk) and T (non-risk). (**B**) In synovium, these alleles differentially bind transcription factors TF1 and TF2; in cartilage, they differentially bind transcription factors TF3 and TF4. (**C**) Differential transcription factor binding at the variant leads to allele-specific methylation of the enhancer. (**D**) This results in quantitative differences in the binding of a common transcription factor (TF5) at the enhancer. (**E**) Low levels of bound TF5 lead to low levels of *COLGALT2* transcription. In synovium, risk allele C is more methylated at the enhancer than non-risk allele T, resulting in less TF5 binding to, and therefore relatively low transcription of, allele C. The opposite is the case in cartilage. The model predicts that for both tissues, decreased enhancer methylation increases *COLGALT2* expression, and *vice versa*.

## Data Availability

Raw data is presented in Additional files 2, 3, 5, 6, 7, 8, and 9.

## Abbreviations

AEI: Allelic expression imbalance
CpG: Cytosine-Guanine dinucleotide
Cas9: CRISPR-associated protein 9
CRISPR: Clustered regularly interspaced short palindromic repeats
dCas9: Deactivated (dead) Cas9
dDNMT3a: Deactivated (dead) DNMT3a
dTET1: Deactivated (dead) TET1
DMR: Differentially methylated region
DNAm: DNA methylation
DNMT3a: DNA (cytosine-5)-methyltransferase 3A
gRNA: Guide RNA
GEO: Gene Expression Omnibus
mQTL: Methylation quantitative trait locus
NHS: National Health Service
OA: Osteoarthritis
QC: Quality control
RT-qPCR: Real-time quantitative polymerase chain reaction
SEM: Standard error of the mean
TET1: Ten-eleven translocation methylcytosine dioxygenase 1
TF: Transcription factor
TPM: Transcripts per million
UCSC: University of California Santa Cruz
UTR: Untranslated region
