# Supplementary figures and images for: Osteoarthritis genetic risk acting on the galactosyltransferase gene COLGALT2 has opposing functional effects in articulating joint tissues
Source: Arthritis Res Ther. 2023 May 19;25:83. doi: 10.1186/s13075-023-03066-y (PMC10197248; doi:10.1186/s13075-023-03066-y)

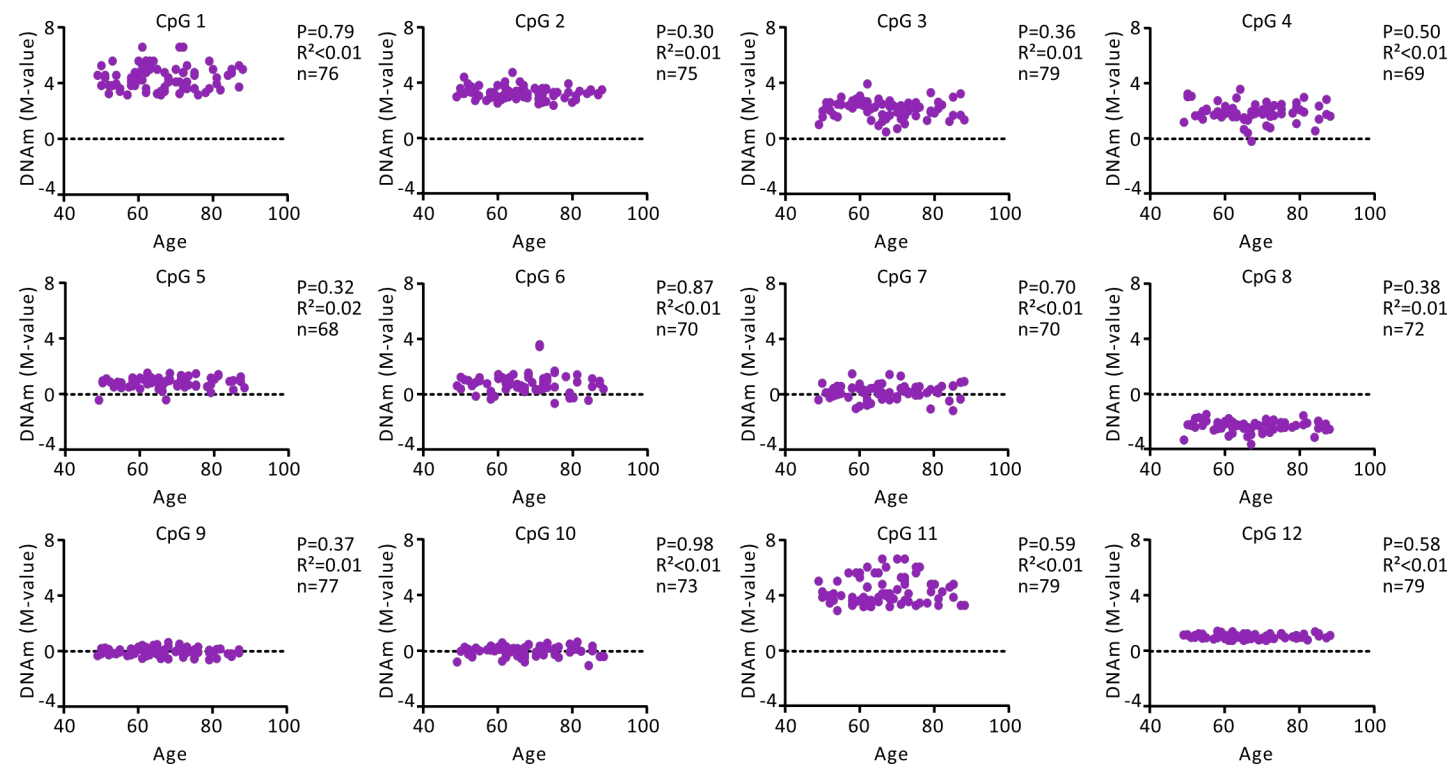

Supplement: Supplementary file 4 — Additional file 4. Age versus methylation. Linear regression was used to test for association between age at surgery inyears and DNA methylation levels at cg18131582 (CpG9) and its 11 flanking CpGs. DNAm, DNA methylation. Each dot is data from one individual. [file 13075_2023_3066_MOESM4_ESM.pdf]

A

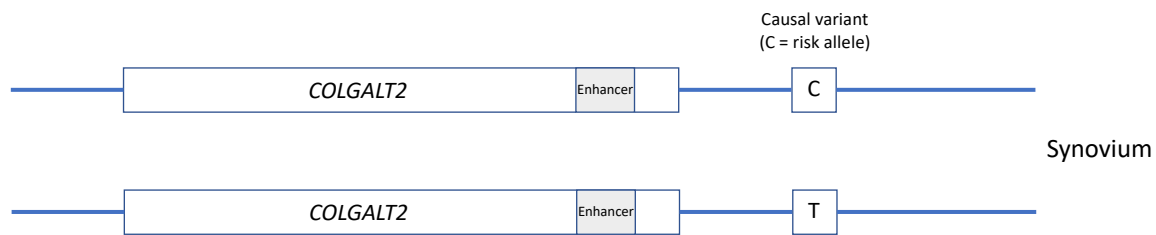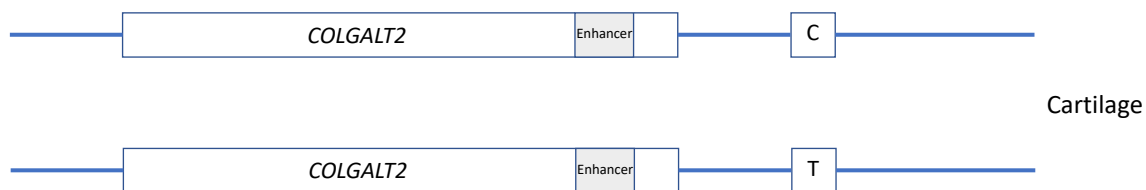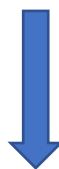

B

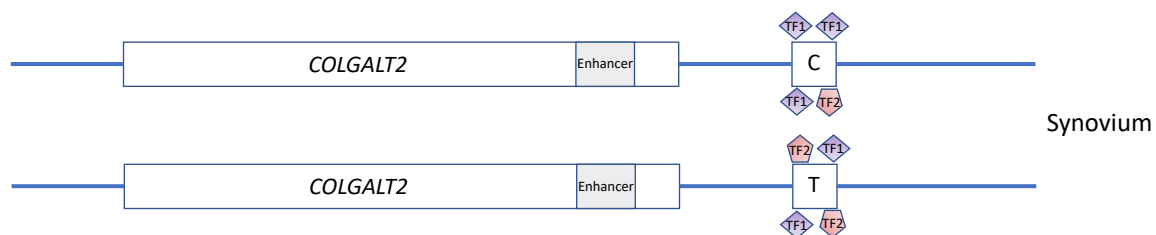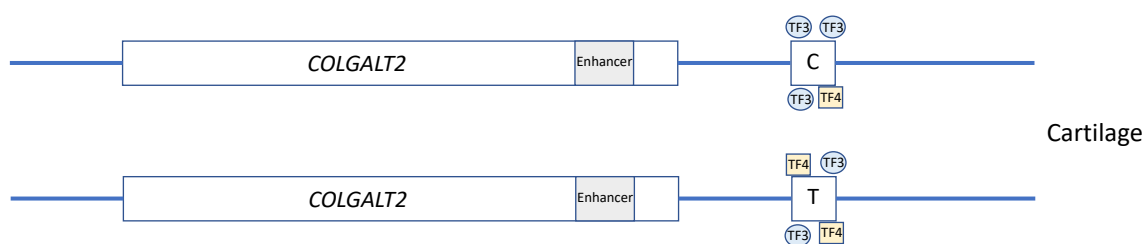

TF1 TF2 = synovium transcription factors  
TF3 TF4 = cartilage transcription factors

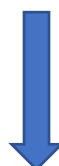

C

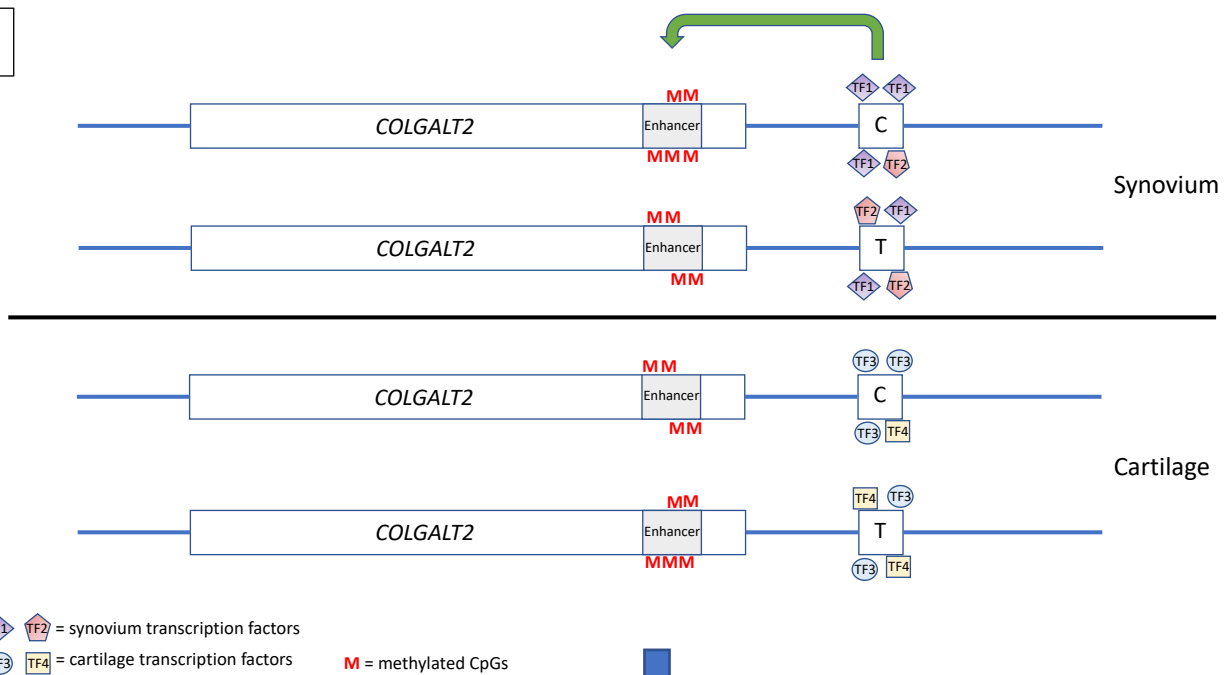

D

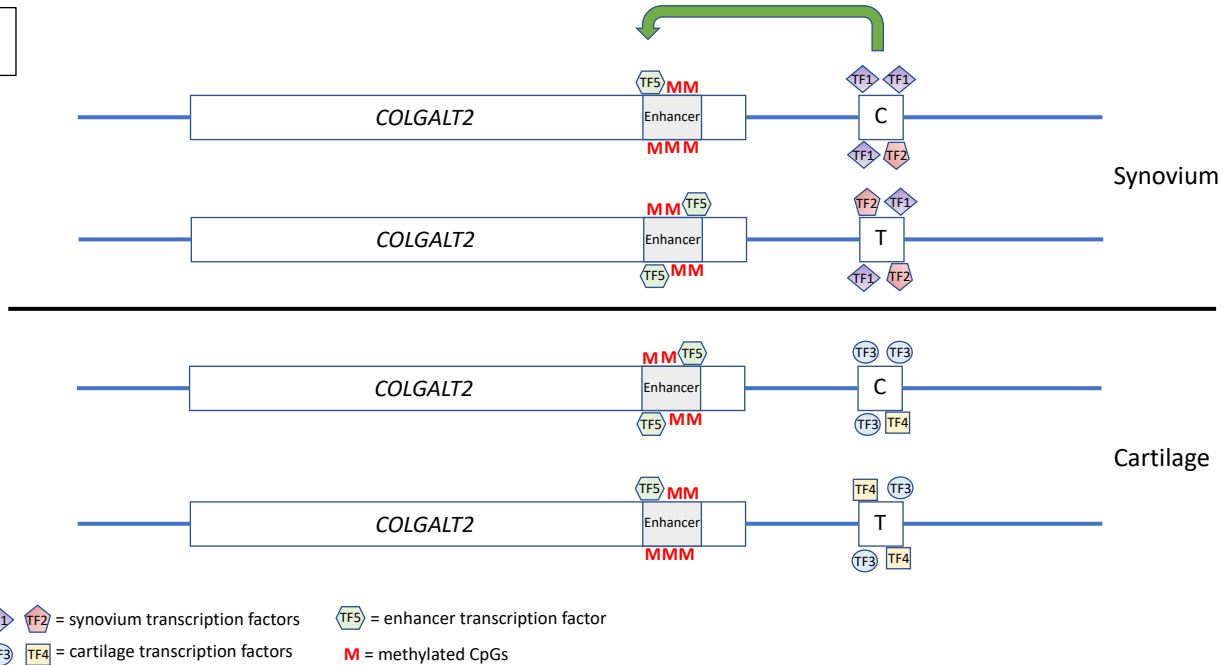

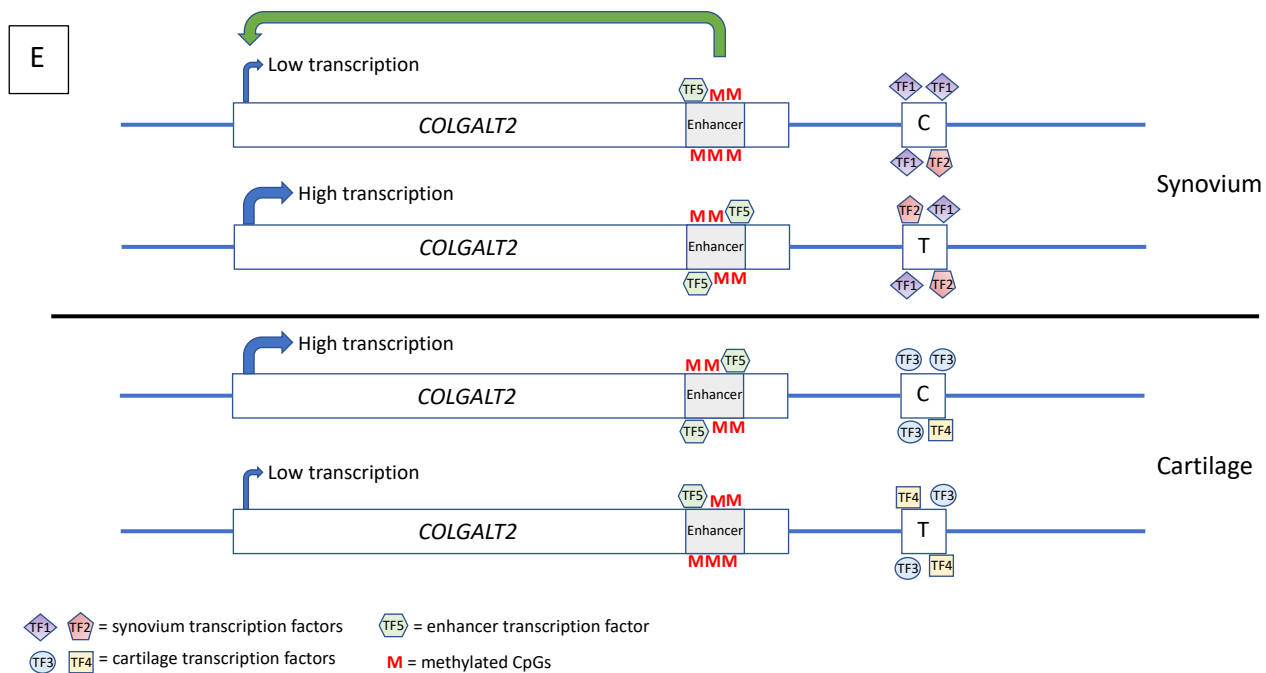

Supplement: Supplementary file 10 — Additional file 10. Flow version of Fig. 7. (A) The causal variant has hypothetical alleles C (risk) and T (non-risk). (B) In synovium, these alleles differentially bind transcription factors TF1 and TF2; in cartilage, they differentially bind transcription factors TF3 and TF4. (C) Differential transcription factor binding at the variant leads to allele-specific methylation of the enhancer. (D) This results in quantitative differences in the binding of a common transcription factor (TF5) at the enhancer. (E) Low levels of bound TF5 lead to low levels of COLGALT2 transcription. In synovium, risk allele C is more methylated at the enhancer than non-risk allele T, resulting in less TF5 binding to, and therefore relatively low transcription of, allele C. The opposite is the case in cartilage. The model predicts that for both tissues, decreased enhancer methylation increases COLGALT2 expression, and vice versa. [file 13075_2023_3066_MOESM10_ESM.pdf]
